# Reference Values for Point-of-Care Echocardiographic Measurements of Preterm Infants in China

**DOI:** 10.3389/fped.2022.894152

**Published:** 2022-06-30

**Authors:** Dan-Fang Lu, Xiao-Mei Tong, Yun-Feng Liu, Hua Zhang

**Affiliations:** ^1^Department of Pediatrics, Peking University Third Hospital, Beijing, China; ^2^Department of Epidemiology Center, Peking University Third Hospital, Beijing, China

**Keywords:** reference values, echocardiography, point-of-care, preterm infant, cardiology

## Abstract

**Background:**

Few studies have examined the reference value of the left ventricular structure and function in preterm infants. This study was designed to establish a point-of-care echocardiographic reference range of left ventricular structure and function based on different gestational age, weight, and body surface area (BSA) for preterm infants within 7 days after birth.

**Methods:**

We retrospectively studied 489 patients with traditional echocardiographic data of left ventricular (LV) M-mode: LV end diastolic dimensions (LVED), LV end systolic dimension (LVES), end-diastolic interventricular septal thickness (IVSd), end diastolic LV posterior wall thickness (LVPWd), left atrial (LA) and aortic root (AO) diameters, and index of LA/AO, LV ejection fraction (LVEF), LV fractional shortening (LVFS), and pulsed wave Doppler: aortic valve flow rate (AV), peak mitral valve flow rate E(MV-E), peak mitral valve flow rate A(MV-A), and MV-E/A. The LV dimensions and the maximum blood flow velocities of the aortic valves and mitral valves according to gestational age, birth weight, and body surface area (BSA) are presented in percentiles tables. Percentile curves of aforesaid four cardiac measurements (LVED, LA diameter (LAD), MV-E, MV-E/A) using the R language Generalized Additive Models for Location, Scale and Shape (GAMLSS) method were developed according to different gestational ages and weights.

**Results:**

Measurements of all cardiac dimensions and Doppler maximum velocities of AV, MV-E, and MV-E/A showed a correlation with gestational age, weight, and BSA. LVED, LAD, MV-E, and MV-E/A showed a trend of increasing values with gestational age and weight on the percentile curves.

**Conclusion:**

The percentile tables and graphs of these point-of-care echocardiographic data can provide reliable reference data for Chinese neonates. Normative values are recommended as a source of reference data for the identification of potentially abnormal echocardiography.

## Introduction

Point-of-care echocardiography is an essential tool in critical care. Its role is emphasized in neonates, as other monitoring techniques may be unavailable (1). Point-of-care echocardiography—-performed by neonatologists in the neonatal intensive care unit (NICU)—-can quickly and dynamically evaluate patent ductus arteriosus, shock, heart failure, pericardial effusion, and persistent pulmonary hypertension in neonates (2–6). This enables neonatologists to make decisions based on the child’s clinical condition, medications, ventilation strategies, and fluid management and formulate a precise treatment plan. This critical heart condition is more likely to occur in premature infants within 7 days of the transition period (7–10). Therefore, point-of-care echocardiography is extremely important in guiding the clinical treatment of critically ill premature infants. In the past 20 years, several large sample studies have suggested normative data related to cardiac chambers and wall thickness in children; however, the age span of the targeted patient population is relatively large, ranging from to 0–18 years (11–14). In recent years, some large sample studies on the heart structure of preterm infants based on body weight and body surface area (BSA) have been conducted, but the weight distribution of preterm infants in these studies was less than 2,000 g (15, 16). One study focused on the normal value of the left ventricular(LV) mass (15), and the other focused on the normal value of the cardiac structure in the patent ductus arteriosus group (16). There are few studies on the reference value of the LV structure and function in premature infants within 7 days of birth (<37 weeks). Therefore, we aimed to establish reference values for LV inner diameter, ventricular wall thickness, and blood flow velocity using two-dimensional (2D), M-mode or Doppler echocardiographic measurements for Chinese neonates, to make a quick and accurate judgment in daily clinical practice.

## Materials and Methods

This retrospective, observational, single-center study of the reports of point-of-care echocardiographic examinations was performed on a population of preterm Chinese infants in the first 7 days of life. Our study was conducted in the Department of Neonatal Intensive Care Unit, Peking University Third Hospital, from March 2017 to February 2020. The study was approved by the Peking University Third Hospital Medical Science Research Ethics Committee (2020114-02), exempted informed consent.

### Patient Data and Definitions

Inclusion criteria were: hemodynamically stable infants with at least one echocardiogram taken within 7 days after birth (including any patient with incomplete data). Exclusion criteria were: (1) Congenital heart disease and/or other congenital malformations; (2) hemodynamic significant patent ductus arteriosus (HSPDA); (3) sepsis; (4) persistent pulmonary hypertension; (5) renal failure; (6) necrotizing enterocolitis, gastrointestinal perforation or intestinal obstruction; (7) severe anemia or shock; (8) smaller or larger than gestational age; and (9) high-parameter invasive ventilation (mean average airway pressure > 8 cmH_2_O [1 cmH_2_O = 0.098 kPa]), and/or inhaled oxygen concentration degree > 0.40.

Hemodynamic significant patent ductus arteriosus was defined as a ductal diameter > 1.5 mm (left to right shunt) and left artrial (LA) diameter/aortic root (AO) > 1.5; or the requirement of positive inotropic drugs, or mechanical ventilation; or if pulmonary edema was present, or gastrointestinal intolerance (17–19).

### Ultrasound Equipment

Echocardiographic examinations were performed with a Mindray M7 scanner produced by Shenzhen Mindray Bio-Medical Electronics Co., (Probe: P12-4S Frequency 4–12 MHZ).

### Echocardiographic Measurements and Clinical Data

**FIGURE 1:**
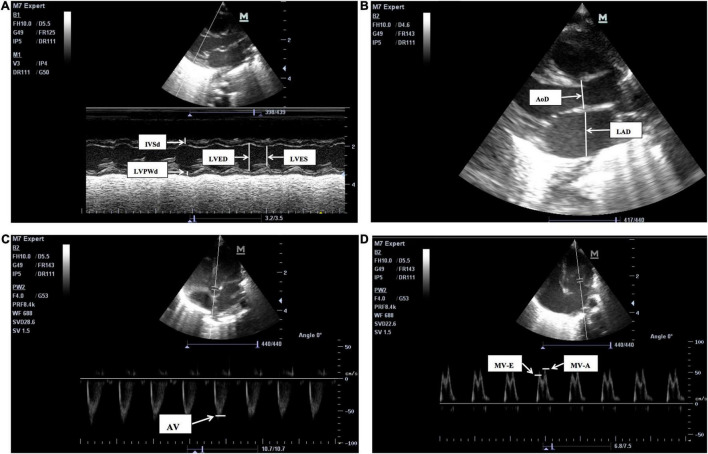
The two-dimensional (2D), M-mode or Doppler echocardiographic measurements pictures for LV structure and function. **(A)** The M-mode echocardiographic measurements for left ventricle and interventricular septum. IVSd, end-diastolic interventricular septum, LVPWd, left ventricular posterior wall end-diastolic thickness, LVED, Left ventricular end-diastolic, LVES, left ventricular end-systolic, LVFS, left ventricular fractional shortening, LVEF, left ventricular ejection fraction. LVFS (%), (LVED-LVES)/LVED × 100, LVEF is calculated by Teichholtz method. **(B)** The 2D measurements of left atrium diameters (LAD) and aortic root diameters (AoD), were taken in end-systole at the level of the aortic valves. LA/AO = LAD/AoD. **(C)** The pulsed wave Doppler measurements for aortic valve flow rate (AV). **(D)** The pulsed wave Doppler measurements for peak mitral valve flow rate E(MV-E), peak mitral valve flow rate A(MV-A), the peak mitral valve flow rate E to A (MV-E/A), MV-E/MV-A.

Echocardiograms were performed by a neonatologist, and examinations were completed by a neonatologist proficient in echocardiography techniques. Instant measurements were reviewed by a cardiologist. Measurements were made over three cardiac cycles, and the mean values were obtained in the standard precordial positions using the published American Child Echocardiography Guide and Standards and American NICU echocardiography Practice Guide (20, 21). The following cardiovascular measurements were obtained from parasternal long-axis views on cross-sectional LV M-mode (Figure 1): LV end-diastolic dimensions (LVED), LV end-systolic dimensions (LVES), end-diastolic interventricular septal thickness (IVSd), end diastolic LV posterior wall thickness (LVPWd), LA, and aortic root (AO) diameters, and index of LA/AO, left ventricular ejection fraction (LVEF), and left ventricular fractional shortening (LVFS). Measurements of LVED, LVES, IVSd, LVPWd, LVEF, and LVFS diameters were taken at the level of the posterior mitral valve leaflet, and measurements of the AO and LA diameters (AOD and LAD) were taken in end-systole at the level of the aortic valves. Maximum blood flow velocities were measured on the aortic and mitral valves and measured from an apical four-chamber and five chamber view using pulsed wave Doppler from a point at the center of the valves (Figure 1):aortic valve flow rate (AV), peak mitral valve flow rate E(MV-E), peak mitral valve flow rate A(MV-A), and MV-E/A. Among these traditional cardiac parameters LVED, LAD, and LA/AO represent the left heart volume load;LVFS and LVEF represent left ventricular systolic function; and MV-E and MV-E/A reflect left ventricular diastolic function. The aforementioned echocardiographic parameters, such as LAD, LA/AO, LVED, and MV-E/A have been examined as predictors of HSPDA (22, 23).

The following clinical variables were noted: antenatal steroids, birth weight, gestational age, body length, fetal birth mode, cause of premature birth, Apgar score, maternal pregnancy complications, and vital data parameters including ventilation mode, mean airway pressure and inhaled oxygen concentration, heart rate, respiratory rate, systolic pressure, diastolic pressure, and mean pressure during measurement.

### Study Group

According to the gestational age (GA), the preterm infants were divided into four groups: <28 weeks, 28–31^+6^ weeks, 32–33^+6^ weeks, and 34–36^+6^ weeks, and also divided into five groups according to birth weight(BW): <1000, 1000–1499, 1500–1999, 2000–2499 and ≥2500 g. Based on BSA, infants were equally divided into seven groups, ranging from 0.07 to 0.20 m^2^.

### Statistical Analysis

Data were analyzed using SPSS (Statistical Package for Social Sciences) version 24.0. BSA was determined using the DuBois and DuBois formula: BSA = 0.20247 × height(m)^0.725^ × weight(kg)^0.425^. Measurements of LVED, LVES, IVSd, LVPWd, LAD, AOD, AV, MV-E, and MV-E/A values were plotted against GA, BW, and BSA. Normality was then tested using the Kolmogorov–Smirnov test, and Spearman’s rank correlation was performed for each parameter. Descriptive statistics were presented as percentiles (3rd, 10th, 25th, 50th, 75th, 90th, and 97th) for the GA, BW, and BSA subgroups. The R language Generalized Additive Models for Location, Scale and Shape (GAMLSS) package was used to draw scatter plots of each measurement in relation to GA and BW, and box-cox transformation and curved lines of centiles (3rd, 10th, 25th, 50th, 75th, 90th, and 97th percentiles) show changes in the four important parameters (LVED, LAD, MV-E and MV-E/A) of GA and BW. A *p*-value less than 0.05 was considered significant.

## Results

Our study included 489 preterm infants with a mean GA of 32 weeks (range: 24–36.7), mean BW of 1,700 g (range: 650–3,180), and mean BSA of 0.13 m^2^ (range: 0.07–0.20 m^2^). The general characteristics of the infants are presented in Table 1.

**TABLE 1:** General characteristics of the preterm babies.

Characteristics	Result
Male: Female(N)	264:225
Length (cm) Median (IQR)	41 (38–44)
SBP (mmHg) Median (IQR)	69 (63–74)
DBP (mmHg) Median (IQR)	37 (32–43)
MBP (mmHg) Median (IQR)	48 (44–53)
Heart rate (times/minute) Median (IQR)	143 (135–153)
Respiratory rate (times per minute) Median (IQR)	40 (35–46)

*IQR, interquartile range.*

In Table 2, the measurements of all cardiac dimensions and Doppler maximum velocities of AV, MV-E, and MV-E/A show a correlation with GA, BW, and BSA (*p* < 0.001). Echocardiographic parameters of LA/AO, LVEF, LVFS, and MV-A show no correlation with GA, BW, and BSA (*p* > 0.05).

**TABLE 2:** Correlation of echocardiographic measurements with GA, BW, and BSA in preterm infants.

Echocardiographic measurements	GA (wk) r (P)	BW (g) r (P)	BSA (m^2^) r (P)
LVED	0.608 (<0.001)	0.609 (<0.001)	0.616 (<0.001)
LVES	0.495 (<0.001)	0.496 (<0.001)	0.505 (<0.001)
IVSd	0.209 (<0.001)	0.275 (<0.001)	0.254 (<0.001)
LVPWd	0.216 (<0.001)	0.288 (<0.001)	0.269 (<0.001)
LAD	0.430 (<0.001)	0.445 (<0.001)	0.437 (<0.001)
AoD	0.481 (<0.001)	0.508 (<0.001)	0.503 (<0.001)
LA/AO	0.07 (0.125)	0.065 (0.154)	0.062 (0.173)
LVEF	−0.021 (0.641)	−0.013(0.641)	−0.019 (0.668)
LVFS	0.022 (0.623)	0.033(0.470)	0.027 (0.550)
AV	0.263 (<0.001)	0.292 (<0.001)	0.278 (<0.001)
MV-A	−0.025 (0.578)	0.049 (0.285)	0.037 (0.418)
MV-E	0.256 (<0.001)	0.261 (<0.001)	0.268 (<0.001)
MV-E/A	0.344 (<0.001)	0.301 (<0.001)	0.312 (<0.001)

*LVED, left ventricular end-diastolic, LVES, left ventricular end-systolic, IVSd, end-diastolic interventricular septum, LVPWd, left ventricular posterior wall end-diastolic thickness, LAD, left atrium diameters, and AoD, aortic root diameters, LA to AO ratio (LA/AO), left ventricular ejection fraction (LVEF), left ventricular fractional shortening (LVFS), aortic valve flow rate (AV), peak mitral valve flow rate E (MV-E), peak mitral valve flow rate A (MV-A), MV-E to MV-A ratio (MV-E/A).*

Tables 3–11 show the nine cardiac measurement distributions as percentiles (3rd, 10th, 25th, 50th, 75th, 90th, and 97th) for each GA, BW, and BSA subgroup. Table 12 shows another four cardiac measurement distributions of 489 babies as percentiles (3rd, 10th, 25th, 50th, 75th, 90th, and 97th) within 7 days after birth.

**TABLE 3:** Left ventricular end-diastolic dimensions (mm) (from 3rd to 97th centiles) in relation to GA, BW, and BSA in preterm infants.

GA (wk)	n	3rd	10th	25th	50th	75th	90th	97th
<28	46	7.41	8.50	10.00	11.00	12.00	13.56	15.35
28–31^+6^	185	9.46	10.06	11.50	12.70	13.95	15.00	16.08
32–33^+6^	140	10.25	12.00	13.10	14.25	15.60	16.67	17.58
34–36^+6^	118	11.56	12.78	14.00	15.10	16.90	18.01	20.03
BW(g)								
<1000	43	7.36	8.74	10.00	11.00	12.00	13.20	14.65
1000–1499	132	9.30	10.00	11.00	12.30	13.20	14.30	15.60
1500–1999	176	10.06	11.90	13.00	14.15	15.40	16.70	17.41
2000–2499	103	11.00	12.22	13.20	15.00	16.40	17.80	19.84
≥2500	35	12.82	13.62	14.00	15.20	16.20	17.52	19.00
BSA(m^2^)								
–0.08	16	7.70	8.61	10.23	11.10	12.90	14.23	–
–0.10	73	8.42	9.38	10.10	11.20	12.35	13.00	14.30
–0.12	103	9.50	10.44	11.50	13.00	14.00	15.00	15.89
–0.14	134	10.21	11.90	13.00	14.00	15.33	16.50	17.58
–0.16	117	10.11	12.42	13.55	14.80	16.25	17.26	18.78
–0.18	39	12.40	12.80	13.90	15.20	16.60	17.80	23.00
–0.20	7	14.30	14.30	14.60	16.00	18.00	–	–

*LVED, left ventricular end-diastolic.*

**TABLE 4:** Left ventricular end-systolic dimensions (mm) (from 3rd to 97th centiles) in relation to GA, BW, and BSA in preterm infants.

GA (wk)	n	3rd	10th	25th	50th	75th	90th	97th
<28	46	4.59	5.24	5.85	7.30	8.00	8.90	12.95
28–31^+6^	185	5.00	6.50	7.20	8.10	9.35	10.24	10.98
32–33^+6^	140	6.05	7.13	8.40	9.20	10.20	11.09	12.08
34–36^+6^	118	6.56	7.50	8.80	10.00	10.93	12.01	13.17
BW(g)								
<1000	43	5.00	5.38	6.00	7.00	8.00	8.90	13.40
1000–1499	132	5.00	6.23	7.20	8.00	8.98	9.87	10.60
1500–1999	176	5.63	7.07	8.40	9.10	10.20	11.00	11.94
2000–2499	103	6.30	7.00	8.50	9.80	10.90	12.00	13.52
≥2500	35	4.26	7.62	8.80	10.00	10.70	11.98	13.00
BSA(m^2^)								
–0.08	16	5.50	5.50	6.13	7.00	8.75	11.50	–
–0.10	73	5.00	5.52	6.55	7.30	8.00	8.86	9.96
–0.12	103	5.07	6.84	7.50	8.30	9.20	10.16	10.80
–0.14	134	5.72	7.00	8.28	9.00	10.23	11.00	12.10
–0.16	117	6.16	7.16	8.70	9.90	10.50	11.62	12.54
–0.18	39	4.54	7.30	8.80	10.30	10.80	12.90	14.92
–0.20	7	9.10	9.10	9.70	10.50	11.00	–	–

*LVES, left ventricular end-systolic.*

**TABLE 5:** End-diastolic interventricular septum dimensions (mm) (from 3rd to 97th centiles) in relation to GA, BW, and BSA in preterm infants.

GA (wk)	n	3rd	10th	25th	50th	75th	90th	97th
<28	46	1.72	1.97	2.30	2.70	3.53	4.26	4.68
28–31^+6^	185	2.00	2.20	2.60	3.00	3.55	4.30	5.47
32–33^+6^	140	2.20	2.50	2.80	3.20	3.60	4.60	5.78
34–36^+6^	118	2.10	2.40	2.70	3.50	4.00	4.60	5.67
BW(g)								
<1000	43	1.90	2.00	2.40	2.70	3.40	4.24	4.70
1000–1499	132	1.80	2.00	2.50	3.00	3.50	4.00	4.30
1500–1999	176	2.00	2.40	2.80	3.15	3.50	4.40	5.61
2000–2499	103	2.21	2.50	3.00	3.50	4.30	5.08	6.09
≥2 500	35	2.40	2.50	2.70	3.00	3.80	4.84	6.33
BSA(m^2^)								
–0.08	16	2.00	2.00	2.28	3.05	3.60	4.15	–
–0.10	73	1.62	2.00	2.35	2.70	3.40	4.20	4.78
–0.12	103	1.91	2.10	2.50	3.00	3.50	4.00	4.30
–0.14	134	2.01	2.50	2.80	3.20	3.73	4.55	5.68
–0.16	117	2.30	2.50	3.00	3.40	4.00	4.70	6.13
–0.18	39	2.10	2.40	2.70	3.20	4.00	5.30	6.08
–0.20	7	2.50	2.50	2.70	3.00	3.50	–	–

*IVSd, end-diastolic interventricular septum.*

**TABLE 6:** Left ventricular posterior wall end-diastolic thickness dimensions (mm) (from 3rd to 97th centiles) in relation to GA, BW, and BSA in preterm infants.

GA (wk)	n	3rd	10th	25th	50th	75th	90th	97th
<28	46	1.42	1.87	2.10	2.40	2.75	3.12	4.12
28–31^+6^	185	1.60	2.00	2.10	2.50	3.00	3.40	4.00
32–33^+6^	140	2.00	2.10	2.40	2.70	3.00	3.50	4.00
34–36^+6^	118	2.00	2.20	2.40	2.85	3.20	3.61	4.13
BW(g)								
<1000	43	1.40	1.68	2.00	2.40	2.90	3.00	4.14
1000–1499	132	1.60	2.00	2.10	2.40	2.90	3.17	3.90
1500–1999	176	2.00	2.00	2.40	2.70	3.00	3.50	4.14
2000–2499	103	2.00	2.20	2.50	2.90	3.40	3.80	4.26
≥2500	35	2.00	2.10	2.40	2.70	3.00	3.50	3.69
BSA(m^2^)								
–0.08	16	1.60	1.60	1.85	2.40	2.88	3.30	–
–0.10	73	1.60	1.94	2.05	2.40	2.90	3.20	4.00
–0.12	103	1.81	2.00	2.10	2.50	3.00	3.20	3.88
–0.14	134	2.00	2.00	2.40	2.70	3.10	3.50	4.29
–0.16	117	2.00	2.20	2.40	2.90	3.20	3.72	4.00
–0.18	39	1.76	2.10	2.40	2.90	3.20	3.50	4.18
–0.20	7	2.10	2.10	2.50	2.60	3.20	–	–

*LVPWd, left ventricular posterior wall end-diastolic thickness.*

**TABLE 7:** Left atrium diameters (mm) (from 3rd to 97th centiles) in relation to GA, BW, and BSA in preterm infants.

GA (wk)	n	3rd	10th	25th	50th	75th	90th	97th
<28	46	4.78	5.00	5.98	7.05	8.80	10.29	11.04
28–31^+6^	185	5.06	5.96	6.65	7.80	8.95	10.00	11.17
32–33^+6^	140	5.92	6.70	7.80	8.60	9.70	10.80	11.55
34–36^+6^	118	5.87	7.09	8.10	9.20	10.05	11.42	12.74
BW(g)								
<1000	43	3.82	4.90	5.90	7.00	8.10	10.30	11.07
1000–1499	132	5.00	5.90	6.40	7.50	8.40	9.50	10.50
1500–1999	176	6.00	6.64	7.80	8.70	9.70	10.80	11.60
2000–2499	103	5.72	7.00	8.00	9.20	10.00	11.22	12.49
≥2500	35	7.00	7.88	8.90	9.20	10.00	12.00	13.14
BSA(m^2^)								
–0.08	16	4.70	4.91	5.58	7.25	9.73	10.92	–
–0.10	73	4.82	5.04	6.00	7.00	8.05	8.92	10.43
–0.12	103	5.40	6.00	6.70	8.00	9.00	10.00	10.93
–0.14	134	6.00	6.45	7.70	8.55	9.70	10.80	11.60
–0.16	117	5.90	7.00	8.00	9.00	10.00	11.32	12.28
–0.18	39	5.94	7.80	8.90	9.20	10.40	11.90	14.16
–0.20	7	7.00	7.00	7.70	10.00	12.00	–	–

*LAD, left atrium diameters.*

**TABLE 8:** Aortic root diameters (mm) (from 3rd to 97th centiles) in relation to GA, BW, and BSA in preterm infants.

GA (wk)	n	3rd	10th	25th	50th	75th	90th	97th
<28	46	4.08	4.40	4.90	5.15	5.83	6.50	7.79
28–31^+6^	185	4.37	5.00	5.40	6.00	6.70	7.00	7.97
32–33^+6^	140	5.00	5.10	6.00	6.50	7.10	7.80	8.28
34–36^+6^	118	5.10	5.69	6.20	7.00	7.50	8.32	9.20
BW(g)								
<1000	43	4.00	4.08	4.60	5.10	5.80	6.50	6.84
1000–1499	132	4.50	5.00	5.30	5.95	6.50	7.00	7.80
1500–1999	176	5.00	5.10	5.90	6.50	7.00	7.63	8.24
2000–2499	103	5.00	5.48	6.20	7.00	7.30	8.26	9.16
≥2500	35	5.40	5.88	6.30	7.10	7.80	8.60	9.18
BSA(m^2^)								
–0.08	16	4.00	4.14	4.93	5.45	5.98	6.29	–
–0.10	73	4.00	4.50	4.95	5.60	6.00	6.60	7.62
–0.12	103	4.82	5.00	5.40	6.00	6.70	7.00	7.50
–0.14	134	5.00	5.10	6.00	6.50	7.03	7.70	8.40
–0.16	117	5.00	5.40	6.00	6.70	7.35	8.02	8.85
–0.18	39	5.40	5.90	6.60	7.00	8.00	8.60	9.92
–0.20	7	6.00	6.00	6.00	6.70	7.50	–	–

*AoD, aortic root diameters.*

**TABLE 9:** Doppler maximum velocities of AV (cm/s) (from 3rd to 97th centiles) in relation to GA, BW, and BSA in preterm infants.

GA (wk)	n	3rd	10th	25th	50th	75th	90th	97th
<28	46	32.00	37.90	46.50	52.80	58.25	68.90	174.89
28–31^+6^	185	30.58	34.60	41.50	52.00	63.50	78.80	92.68
32–33^+6^	140	36.00	42.10	50.50	59.50	71.00	78.90	88.00
34–36^+6^	118	36.57	41.00	51.00	61.50	71.25	82.10	93.72
BW(g)								
<1000	43	26.56	32.40	39.00	50.00	60.00	69.80	192.20
1000–1499	132	30.00	36.30	42.00	50.00	59.75	71.00	80.03
1500–1999	176	34.62	41.00	50.00	59.50	72.00	82.00	94.38
2000–2499	103	36.12	40.00	52.00	63.00	71.00	83.60	96.88
≥2500	35	33.24	41.60	50.00	61.00	70.00	76.40	86.68
BSA(m^2^)								
–0.08	16	24.00	29.60	34.75	52.80	59.00	123.20	–
–0.10	73	30.22	34.00	42.50	50.00	59.50	67.00	75.56
–0.12	103	32.00	36.40	41.00	50.00	61.00	72.60	82.64
–0.14	134	34.10	42.50	52.00	61.00	76.25	84.50	100.75
–0.16	117	36.00	40.00	50.00	60.00	71.00	82.00	96.00
–0.18	39	34.40	46.00	55.00	63.00	70.00	78.00	87.80
–0.20	7	36.00	36.00	41.00	66.00	74.00	–	–

*AV, aortic valve flow rate.*

**TABLE 10:** Doppler maximum velocities of MV-E (cm/s) (from 3rd to 97th centiles) in relation to GA, BW and BSA in preterm infants.

GA (wk)	n	3rd	10th	25th	50th	75th	90th	97th
<28	46	20.00	23.00	30.00	37.00	47.25	68.50	94.36
28–31^+6^	185	20.00	22.00	26.50	33.00	40.00	50.00	58.84
32–33^+6^	140	22.00	28.10	35.00	40.00	45.75	54.90	63.00
34–36^+6^	118	23.00	27.00	34.00	41.00	48.25	59.10	70.44
BW(g)								
<1000	43	17.96	20.00	23.00	32.00	42.00	59.20	94.72
1000–1499	132	20.00	22.00	25.00	32.00	41.00	50.00	57.01
1500–1999	176	24.00	27.00	33.00	38.50	45.00	55.00	63.69
2000–2499	103	20.12	27.00	36.00	40.00	46.00	58.60	67.00
≥2500	35	20.40	25.60	34.00	43.00	48.00	56.00	81.32
BSA(m^2^)								
–0.08	16	20.00	20.00	23.50	34.00	46.50	93.20	–
–0.10	73	20.00	22.00	24.50	32.00	41.00	51.20	57.00
–0.12	103	20.12	22.40	27.00	33.00	41.00	51.80	62.88
–0.14	134	23.05	26.50	32.00	38.00	45.00	55.00	65.90
–0.16	117	20.00	27.00	33.50	40.00	45.00	56.20	62.00
–0.18	39	21.40	37.00	41.00	45.00	54.00	63.00	81.40
–0.20	7	25.00	25.00	26.00	33.00	45.00	–	–

*MV-E, peak mitral valve flow rate E.*

**TABLE 11:** Doppler maximum velocities of MV-E/A (from 3rd to 97th centiles) in relation to GA, BW, and BSA in preterm infants.

GA (wk)	n	3rd	10th	25th	50th	75th	90th	97th
<28	37	0.49	0.55	0.60	0.70	0.80	0.85	0.92
28–31^+6^	185	0.50	0.57	0.66	0.73	0.81	1.02	1.31
32–33^+6^	140	0.57	0.61	0.69	0.78	0.94	1.20	1.46
34–36^+6^	118	0.59	0.66	0.74	0.84	1.22	1.37	1.85
BW(g)								
<1000	37	0.43	0.50	0.62	0.68	0.77	0.87	1.11
1000–1499	129	0.51	0.56	0.63	0.73	0.83	1.06	1.40
1500–1999	176	0.58	0.63	0.70	0.76	0.90	1.21	1.54
2000–2499	103	0.57	0.65	0.71	0.80	1.10	1.34	1.73
≥2500	35	0.48	0.58	0.73	0.87	1.14	1.31	2.36
BSA(m^2^)								
–0.08	13	0.49	0.52	0.61	0.66	0.77	0.94	–
–0.10	68	0.46	0.54	0.63	0.72	0.80	0.94	1.58
–0.12	102	0.51	0.58	0.63	0.73	0.85	1.09	1.39
–0.14	134	0.57	0.62	0.70	0.76	0.86	1.19	1.41
–0.16	117	0.56	0.65	0.70	0.78	0.94	1.33	1.75
–0.18	39	0.58	0.68	0.80	1.08	1.23	1.33	2.81
–0.20	7	0.54	0.54	0.71	0.80	1.15	–	–

*MV-E/A, MV-E to MV-A ratio.*

**TABLE 12:** Percentiles of four cardiac parameters within seven days of 489 preterm infants.

Cardiac parameters	n	3rd	10th	25th	50th	75th	90th	97th
LA/AO	489	0.89	1.00	1.14	1.33	1.52	1.67	1.87
LVEF	489	55.00	60.00	62.00	67.00	73.00	79.00	84.30
LVFS	489	26.00	29.00	31.00	34.00	38.00	44.00	50.00
MV-A	480	27.43	32.00	38.00	47.00	54.00	62.00	70.00

*Left atrium diameters to aortic root diameters ratio (LA/AO), left ventricular ejection fraction (LVEF), left ventricular fractional shortening (LVFS), peak mitral valve flow rate A (MV-A).*

**FIGURE 2:**
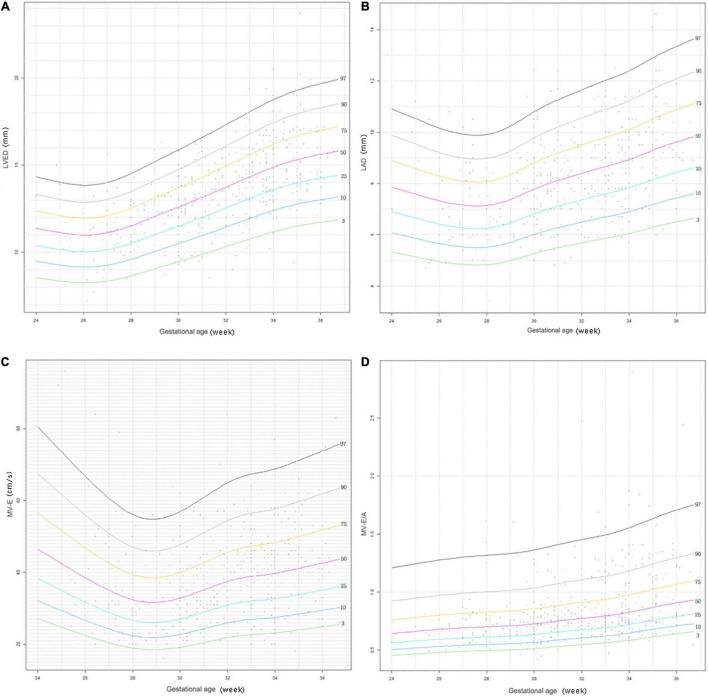
Reference centile curve for 489 preterm infants generated using R language GAMLSS method for **(A)** left ventricular end-diastolic (LVED), **(B)** left atrium diameter (LAD), **(C)** the peak mitral valve flow rate E (MV-E), and **(D)** the peak mitral valve flow rate E to A (MV-E/A) normalized by gestational age within 7 days of birth.

**FIGURE 3:**
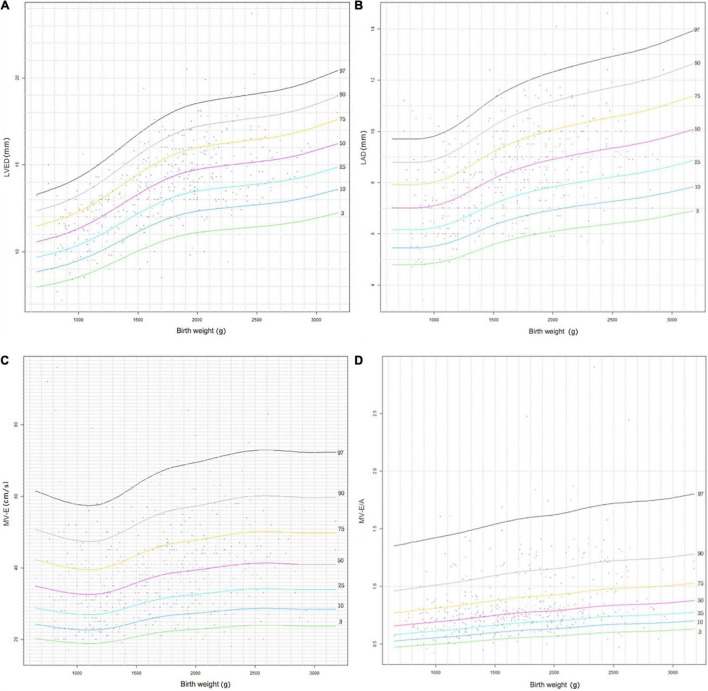
Reference centile curve for 489 preterm infants generated using R language GAMLSS method for **(A)** left ventricular end-diastolic (LVED), **(B)** left atrium diameter (LAD), **(C)** the peak mitral valve flow rate E (MV-E), and **(D)** the peak mitral valve flow rate E to A (MV-E/A) normalized by birth weight within 7 days of birth.

Figures 2, 3 show four important cardiac measurements: percentile curves with data points for all 489 reference patients compared with their GA and BW, respectively. Percentile lines correspond to the 3rd, 10th, 25th, 50th, 75th, 90th, and 97th percentiles.

## Discussion

We present reference ranges of the left cardiac dimensions, mitral and aortic valve maximum blood flow velocities for a complete population of preterm infants. The structure and function of the heart are considered to be related to race (24, 25). Lopez et al. (26) collected data from healthy non-obese children ≤ 18 years of age with a normal echocardiogram from 19 centers and studied the Z scores for common measurements adjusted for body surface area (BSA). They found that race had a small effect on the Z scores. Nevertheless, this study did not include preterm babies. Therefore, it is of great significance to establish a reference range for the heart size and peak valve flow rate of preterm infants during the perinatal period in our own country, which can help determine echocardiographic measurements of a particular patient as normal or abnormal.

Cardiac point of care ultrasound (POCUS) is increasingly being utilized in neonatal intensive care units for providing information in real time to aid clinical decision making. Cardiac POCUS refers to a basic, time-sensitive, and focused diagnostic echocardiographic assessment, including Hemodynamic assessment, HSPDA, and PPHN. In this study, cardiac ultrasonography was performed by neonatologists with formal cardiac ultrasound training. Reference values of LVED, LAD, LVEF, LVFS, MV E/A, and LA/AO, measured in preterm infants within 7 days are helpful for judging left ventricular volume load, left ventricular systolic and diastolic function, and HSPDA, during cardiac POCUS.

The mean gestational age of the infants included in this study was 32 weeks. It is likely that extremely preterm infants under 32 weeks were more likely to develop systemic comorbidities such as HSPDA and PPHN within 7 days of birth, and were therefore excluded from the study.

Studies have found that heart size is positively correlated with GA, body weight or BSA of the newborn. The timings in previous studies were within 0, 7, and 28 days of the newborn’s birth and within 36 weeks of the postmenstrual age of preterm babies (13, 16, 27, 28) and are consistent with our findings. Therefore, for newborns, the heart size corresponding to each index can be used as a reference value. There are few research studies on the reference value of heart valve peak flow velocity. Twenty years ago, Skelton et al. (27) found that the peak flow velocities of the mitral valve, tricuspid valve, aorta, and pulmonary artery had no correlation with GA and BW; however, a recent study found that the MV-E/A value of full-term infants at the age of 4–7 days is higher than that at birth (29). Our study found that AV, MV-E, and MV-E/A were positively correlated with GA, BW, and BSA, which may be related to the small sample size of the previous study. This is consistent with recent research results and conforms to the law of continuous development and maturity of left myocardial function with age; based on the above rules, to establish a more comprehensive normal range of echocardiographic measurements in preterm infants, this study lists percentile tables based on GA, BW, and BSA. For a more intuitive and convenient reference, this study lists percentile curves of four important echocardiographic parameters based on GA and BW.

We found that the four indexes of echocardiography including LA/AO, LVEF, LVFS, and MV-A were not correlated with GA, BW, and BSA within 7 days of preterm infants. These four indexes are relatively independent and stable in the early postnatal period. LA/AO is currently the most commonly used method for predicting HSPDA. At present, many studies have suggested that HSPDA may occur when LA/AO ≥ 1.5 (17, 30). This study found that after excluding HSPDA, LA/AO in premature infants within 7 days of age, the median (interquartile range, IQR)is 1.33 (1.14–1.52). Therefore, when the LA/AO value is greater than the 75th percentile of the reference value, it is necessary to be alerted to the occurrence of HSPDA in premature infants. Regardless of adults or children, LVEF and LVFS are considered the most commonly used simple indicators to measure left ventricular systolic function (31, 32). This study found that neither of them changes with GA, BW, and BSA, which is consistent with the findings of a full-term neonatal heart reference value study (12). This shows that in the postnatal cycle transition period, the LV systolic function of premature infants maintained a relatively stable level, and the median (IQR) was 67 (62–73) and 34 (31–38), respectively. MV-A is the maximum peak flow velocity in the late diastole of the left ventricle, representing the contraction of the left atrium, and is one of the indicators reflecting the diastolic function of the left ventricle (32). This study found that the value of MV-A within 7 days of birth of preterm infants was not correlated with GA, BW, and BSA at birth, indicating that the left atrial contractility changes little in the early postnatal period, which may be related to the immature LV diastolic function of preterm infants.

The LVED and LAD indices are important indicators that reflect left ventricular volume load. It can be seen that LVED and LAD display a gradual increasing trend with increase in GA and BW in the centile curves (Figures 2, 3). The parameters have corresponding reference values for different gestational ages and birth weights. Therefore, the reference range of LVED and LAD in this study is helpful to judge whether left heart overload is present or not in preterm infants within 7 days of birth.

The indices of MV-E and MV-E/A are important indicators of LV diastolic function. A study on the LV function of the fetus at 20–36^+6^ weeks found that MV-E and MV-E/A of the fetus increased with GA (33). A meta-analysis of adults also found that MV-E/A increased with age (34). This study also found that MV-E and MV-E/A increased slightly with increase in GA and BW of preterm infants (Figures 2, 3). In addition, the median MV-E/A value of preterm infants with different gestational ages and birth weights within 7 days of birth was less than 1.00. Compared with full-term infants, MV-E/A decreased significantly at 28 days after birth in preterm infants (35). This shows that the LV diastolic function of premature infants in the early postnatal period is not yet mature. Therefore, the reference range of MV-E and MV-E/A in this study is helpful in determining whether the LV diastolic function of premature infants within 7 days of birth is abnormal.

The reference range of the percentile tables of other cardiac diameters, wall thicknesses, and Doppler blood flow parameters are helpful in judging normal or abnormal hearts.

### Strengths and Limitations

We included a large sample of point-of-care echocardiographic data to establish the normal value of echocardiography of preterm infants from multiple angles. As a limitation, this was a retrospective, single-center study, and the time that each baby underwent echocardiography was not uniform. In this study, premature infants with stable circulatory conditions were selected as much as possible to reduce data deviation caused by time inconsistency. Finally, although 2D point-of-care echocardiography is important for cardiac structural and functional monitoring in preterm infants, we need to look at the overall clinical picture and also caveats about very limited data on three-dimensional functional assessments which are possible in older children and adults.

## Conclusion

Data from this large study in preterm infants provide reference values of LV structure and function based on different GA, BW, and BSA for preterm infants within 7 days of birth. This data could prove to be invaluable in helping neonatologists to judge normal or abnormal hearts in time and make rapid and correct clinical decisions for critically ill premature infants in the cardiopulmonary transition period. We recommending the involvement of more centers and an intra-group difference analysis in prospective studies.

## Data Availability Statement

The raw data supporting the conclusions of this article will be made available by the authors, without undue reservation.

## Ethics Statement

The studies involving human participants were reviewed and approved by the Peking University Third Hospital Medical Science Research Ethics Committee. Written informed consent from the participants’ legal guardian/next of kin was not required to participate in this study in accordance with the national legislation and the institutional requirements.

## Author Contributions

D-FL: data collection, analysis, interpretation, literature search, and manuscript writing. X-MT and Y-FL: supervision, study design, and interpretation. HZ: data analysis and plotting. All authors contributed to the article and approved the submitted version.

## Conflict of Interest

The authors declare that the research was conducted in the absence of any commercial or financial relationships that could be construed as a potential conflict of interest.

## Publisher’s Note

All claims expressed in this article are solely those of the authors and do not necessarily represent those of their affiliated organizations, or those of the publisher, the editors and the reviewers. Any product that may be evaluated in this article, or claim that may be made by its manufacturer, is not guaranteed or endorsed by the publisher.
